# Exosome Released FZD10 Increases Ki-67 Expression *via* Phospho-ERK1/2 in Colorectal and Gastric Cancer

**DOI:** 10.3389/fonc.2021.730093

**Published:** 2021-09-23

**Authors:** Maria Principia Scavo, Federica Rizzi, Nicoletta Depalo, Raffaele Armentano, Sergio Coletta, Grazia Serino, Elisabetta Fanizza, Pasqua Letizia Pesole, Alessandra Cervellera, Nicola Carella, Maria Lucia Curri, Gianluigi Giannelli

**Affiliations:** ^1^ Personalized Medicine Laboratory, National Institute of Gastroenterology “S. De Bellis” Research Hospital, Bari, Italy; ^2^ University of Bari “A. Moro,” Chemistry Department, Bari, Italy; ^3^ Institute for Chemical–Physical Processes (IPCF)–National Research Council Secondary Site (CNR SS) Bari, Bari, Italy; ^4^ Department of Pathology, National Institute of Gastroenterology “S. de Bellis,” Research Hospital, Bari, Italy; ^5^ Experimental Immunopathology Laboratory, National Institute of Gastroenterology “S. de Bellis,” Research Hospital, Bari, Italy; ^6^ Laboratory of Clinical Pathology, National Institute of Gastroenterology, “S de Bellis” Research Hospital, Bari, Italy; ^7^ Scientific Direction, National Institute of Gastroenterology “S. De Bellis” Research Hospital, Bari, Italy

**Keywords:** FZD10, MAPK/ERK, Ki-67, exosomes, colorectal cancer, gastric cancer

## Abstract

Frizzled (FZD) proteins are primary receptors for Wnt signaling that activates the mitogen-activated protein kinase (MAPK) pathways. Dysfunction of Wnt signals with consequently abnormal activation of MAPK3 pathways was found in colorectal cancer (CRC) and gastric cancer (GC). Upregulation of FZD10 protein, localized in the exosomes isolated from plasma of CRC and GC patients, was associated with a poor prognosis. Herein, the expression levels of circulating FZD10 were found to be strongly correlated to their expression levels in the corresponding tissues in CRC and GC patients. Bioinformatic prediction revealed a link between FZD10 and Ki-67 through MAPK3. In both CRC and GC tissues, pERK1/2 levels were significantly increased at more advanced disease stages, and pERK1/2 and Ki-67 were correlated. Silencing of FZD10 in CRC and GC cells resulted in a significant reduction of pERK1/2 and Ki-67 expression, while subsequent treatment with exogenous exosomes partially restored their expression levels. The strong correlation between the expression of Ki-67 in tissues and of FZD10 in exosomes suggests that the exosome-delivered FZD10 may be a promising novel prognostic and diagnostic biomarker for CRC and GC.

## Introduction

Frizzled (FZD) proteins, a family of seven-transmembrane-span proteins with topological homology to G protein-coupled receptors, are involved in the canonical β-catenin and noncanonical Wnt signaling pathways. They regulate several cell processes, such as migration, polarity, neural patterning, and organogenesis during embryonic development (1). In addition to the canonical and noncanonical pathways, Wnt pathways have also been suggested to intersect with other key pathways, including the mitogen-activated protein kinase (MAPK), implicated in cell development, transformation, and apoptosis, as well as to stimulate intracellular proteins or small molecules that act as signaling intermediates (2, 3). Therefore, an intricate signaling network is established as a consequence of the complex cross-cascade interactions among the major signaling pathways. Dysregulation of Wnt signals and alterations in the Wnt cascade proteins and molecular intermediates can induce the onset and progression of many different cancers as well as of alterations in embryo development (4, 5). Mutations and aberrant activation of MAPK pathways were also found to promote cancer progression and spread (6, 7). In particular, experimental evidence showed that variations in MAPK3 influence colorectal cancer (CRC) risk and survival after diagnosis (8).

The Wnt signals are activated by interaction of Wnt proteins with their receptors on the cell surface. FZD proteins act as primary receptors for Wnt signaling pathways, since Wnt ligands are able to bind their N-terminal extracellular cysteine-rich domain (9, 10). Among the 10 isoforms of FZD proteins, the FZD10, a specific protein belonging to this family, involved in canonical and noncanonical Wnt pathways, still remains the most enigmatic (11, 12). The FZD10 expression is very low or absent in developed healthy vital organs. Upregulation of FZD10 expression has been reported in synovial sarcomas and central nervous system (CNS) angiogenesis and in primary CRC (13) and gastric cancer (GC) (14), but the precise biological behavior of FZD10 in regard to tumorigenesis is still unclear (15–18). In this context, our previous study, performed on tissues of patients affected by CRC and GC, highlighted a strong correlation between the expression level and cell localization of FZD10, occurring as a function of different tumor stages (19, 20). FZD10 has been reported to be specifically localized in the exosomes of CRC and GC patients, indicating that this protein may play a crucial biological role in human cancers of the gastrointestinal tract and may be a potential biomarker in the management of CRC and GC (21). On the other hand, a significant number of studies evidenced that tumor-derived exosomes are also actively involved in the carcinogenesis and metastatic dissemination in many types of cancers (22). Exosomes are extracellular nanovesicles ranging from 30 to 150 nm in size; they are released by cells in the extracellular space through the cytoplasmic membranes in both healthy and pathological conditions. They can be isolated starting from different biological fluids, such as blood, saliva, cerebrospinal or amniotic fluid, and urine (23, 24). Exosomes play a key role in intercellular communication, being natural vehicles for different bioactive constituents, such as lipids, proteins, and nucleic acids. In particular, tumor-derived exosomes can transfer their oncogenic components into the recipient cells, thus reprogramming them. This has significant implications on cancer progression (25), and indeed, in the FZD10-mRNA-silenced cells, the viability was restored (26). We recently reported that activation of the epithelial–mesenchymal transition (EMT) in normal colon epithelial cells (HCEC-1CT) is triggered by FZD10-delivering exosomes extracted from CRC cell lines (27).

The exosomes released by CRC cells were found to increase the phosphorylation of extracellular signal-regulated protein kinase (ERK) in surrounding cells, consequently activating MAPK intracellular signaling and promoting tumor growth (26, 28, 29).

Against this background, this study aims to provide new insights on the role of FZD10 in CRC and GC progression. The correlation between FDZ10 expression levels in plasma-derived exosomes from CRC and GC patients at different TNM stages and the expression levels of Ki-67, a well-established clinical marker of cell proliferation and cancer aggressiveness, in the corresponding tissues from colon or stomach were investigated. The final goal is to take a further step the validation of the FDZ10 as a novel diagnostic and prognostic biomarker for CRC and GC.

## Materials and Methods

### Plasma and Tissue Collection From Colon and Gastric Cancer Patients

In total, 66 subjects were enrolled in this study (**Table 1**), including 26 patients affected by CRC (16 M/10F: age 69.53 ± 9.34 years), 20 patients affected by GC (11M/9F: age 71.92 ± 11.99 years), and 20 healthy donors (10 M/10 F: age 50.00 ± 8.67). Patients with sporadic adenocarcinoma of the colon or stomach, without any mutations and older than 50 years, were enrolled, including women in menopause for at least 2 years. Non-steroidal anti-inflammatory drugs were not being taken by any patient at the time of diagnosis. Colon and gastric tissues and plasma were obtained from patients who underwent surgery or colonoscopy for CRC and surgery or gastroscopy for GC. The definitive diagnosis was made at the Pathological Anatomy Department of the National Institute of Gastroenterology at “S. de Bellis” Research Hospital. Tissue samples were selected by using morphological and histological criteria for each pathological stage (according to TNM system) to obtain: A) colon cancer and normal tissue and B) stomach cancer and normal tissue. Each patient signed an informed consent before entering the study.

**Table 1:** Number of patients, cancer type [colorectal cancer (CRC) or gastric cancer (GC)], age, sex, TNM stage, and average of number of metastatic lymph nodes in each enrolled group of patients.

Number of patients	Cancer	Age	Sex	TNM stage	Lymph node metastatis (AVG)
**5**	CRC	43 ± 18	3F/2M	T1N0Mx	0
**4**	CRC	70 ± 20	2F/2M	T2N0Mx	0
**5**	CRC	69 ± 10	2F/3M	T3N0Mx	0
**1**	CRC	75	F	T3N1MX	3/12
**1**	CRC	82	M	T3N2Mx	4/20
**2**	CRC	72 ± 6	2M	T4N0Mx	0
**3**	CRC	60 ± 13	1F/2M	T4N1M1	2.5/35
**5**	CRC	59 ± 19	1F/4M	T4N2Mx	7.2/23
**2**	GC	76 ± 10	2M	T1N0Mx	1/26
**2**	GC	60 ± 5	1F/1M	T2N0Mx	8/12
**1**	GC	57	M	T2N1Mx	5/25
**1**	GC	85	M	T2N2Mx	0
**2**	GC	56 ± 15	1F/1M	T3N0Mx	0
**1**	GC	72	M	T4N0Mx	0
**2**	GC	75 ± 7	1F/1M	T4N1Mx	4/25
**2**	GC	50 ± 36	1F/1M	T4N2Mx	3.5/19
**6**	GC	64 ± 20	2F/5M	T4N3Mx	9.5/37.5

### Exosome Isolation

Plasma specimens from all the subjects were processed for exosome extraction, following a previously reported procedure (21, 22). After the exosome extraction, for each sample, 50 µl of exosome suspension was immediately processed for dynamic light scattering (DLS) and transmission electron microscopy (TEM) characterization as previously reported (**Figure S1, Supplementary Material**) (21, 22), while the remaining sample was stored at -80°C until the protein extraction was carried out. Isolation of exosomes from the culture medium of the two untreated metastatic cell lines, SW620 and N87, for the FDZ10-mRNA silencing experiments, was performed according to a previously reported procedure (**Figure S2, Supplementary Material**) (26).

### Quantification of Proteins Extracted From the Plasma-Derived Exosomes and Western Blotting

After isolation, all exosome samples (derived from plasma) were homogenized by using 1X radioimmunoprecipitation buffer (RIPA; Cell Signaling Technology, Danvers, MA, USA) added with protease inhibitors (Amresco, Solon, OH, USA). The resulting mixture was kept on ice for 30 min; every 5 min, the vials were vortex and replaced on ice. After 30 min, the mixture composed of exosomes and buffer was centrifuged at 15,000 g for 30 min at 4°C, and the supernatant containing the total proteins was used for the Western blotting analysis. The protein amount in the homogenates was measured by means of the Bradford assay (Bio-Rad Hercules, CA, USA).

For each sample, the same protein amount (20 µg) was used to perform the Western blotting, as previously described (21). Briefly, 20 µg of the protein content, after its mixing with reducing Laemmli buffer, was loaded on 4%–15% Tris-glycine sodium dodecyl sulfate-polyacrylamide gels (Bio-Rad, Hercules, CA, USA). After electrophoresis, the separated proteins are blotted onto nitrocellulose membranes (Bio-Rad, Hercules, CA, USA) using a Trans-blot system (Bio-Rad, Hercules, CA, USA) and then treated with 5% non-fat milk (Bio-Rad, Hercules, CA, USA) in Tris-buffered saline supplemented with 0.05% Tween-20 (TBS-T) for 2 h. Subsequently, the primary antibodies, anti-FZD10 (1:400; Abcam, Cambridge, UK), anti-ERK1/2 (1:1,000; Abcam, Cambridge, UK), anti-p-ERK1/2 (1:1,000; Abcam, Cambridge, UK), anti-ALIX (1:1,000; Abcam, Cambridge, UK), and anti-HSP-70 (1:1,000; Abcam, Cambridge, UK) were used for hybridization, performed overnight at 4°C. Subsequently, the membranes were incubated with the corresponding horseradish peroxidase (HRP)-conjugated secondary antibodies for 1 h at room temperature and then washed in TBS-T. An enhanced chemiluminescence kit (Bio-Rad, Hercules, CA, USA) was used. A Chemidoc XRS+ (Bio-Rad, Hercules, CA, USA) was used to observe the chemiluminescence signals from proteins. The images were analyzed by using Image Lab 5.2.1 software.

### Tissue Process and Immunofluorescence Specimen Analysis

For the pathology assessment, 4-µm-thick sections of each type of tissue (colon and stomach) were processed for histology and stained with hematoxylin–eosin (H&E) staining. After tissue classification of each specimen, the immunofluorescence was assessed for CRC and GC and performed for all samples for FZD10 ERK1/2, p-ERK1/2, and Ki-67 proteins. To evaluate the protein expression, rabbit polyclonal anti-FZD10 (1:200; Abcam, Cambridge, UK), rabbit anti-ERK1/2 (1:200; Abcam, Cambridge, UK), mouse anti-p-ERK1/2 (1:200; Abcam, Cambridge, UK), and Ki-67 Mib-1 Clone, mouse monoclonal antibody (DAKO) were used. Briefly, after deparaffinization and antigen retrieval (citrate buffer at pH 6.0 in a water bath at 98°C for 30 min), slides were treated with a solution of 0.5% of Triton X-100 in phosphate buffer saline (PBS 1× pH 7.4) for 10 min at room temperature. Before the incubation with the primary antibody, nonspecific sites were blocked by incubation with fetal bovine serum (FBS) 5% in PBS solution for 1 h, and then the slides were incubated with primary antibodies (diluted respectively 1:200 with PBS and 5% FBS, respectively) overnight at 4°C. Subsequently, all slides were treated with the secondary antibodies Alexa anti-Rabbit 488 (Invitrogen) or Alexa anti-mouse 488 (Invitrogen), both at an absorption maximum wavelength (λ_abs,max_) of 495 nm and emission maximum wavelength (λ_emi,max_) of 517 nm and incubated at room temperature for 1 h, washed in PBS, and mounted with Prolong Gold Antifade reagent containing 4′,6-diamidino-2-phenylindole (DAPI) for nuclear counterstaining. Negative controls were included in each staining run and as negative control; the primary antibody was omitted and replaced by PBS 1× pH 7.4. Specimens were acquired by a pathologist with Eclipse Ti2 confocal microscope by Nikon at ×20 magnification. The images were processed for signal quantification with ImageJ. Staining of all proteins was assessed by counting the number of pixels (%) of immunoreactive tumor cells in more than 10 tumor areas at ×20 magnification. Immunofluorescence assessments were always made independently by two investigators.

### Immunofluorescence Staining of FDZ10, ERK, p-ERK, and Ki-67 in Silenced Cells

The cell lines, namely, N87 and SW620, were seeded onto six-well plates at a density of 5 × 10^4^ cells/well at 37°C. After 24 h, the two cancer cell lines were silenced with the si-PORT-NeoFX transfection agent (Thermo Fisher Scientific) and silencer select FDZ10-siRNA (Thermo Fisher Scientific), according to the previously published protocol (26). Untreated adherent cells were trypsinized and diluted in normal growth medium for 30 min before transfection and replaced at in incubator at 37°C, while transfection complexes were prepared in sterile tubes. The siPOR-NeoFX transfection agent (0.5 µl) was diluted in Opti-MEM I (10 µl) and incubated for 10 min at room temperature. Silencer FDZ10-siRNA was also diluted in Opti-MEM medium at room temperature to a final concentration of 5 nM. The transfection complexes were prepared by mixing the diluted solution of siPORT-NeoFX transfection agent and FDZ10-siRNA, incubated for 10 min at room temperature, and then used to overlay the cells. The culture plates were gently slanted to obtain homogeneous mixing. After 96 h incubation with the transfection complex, each FDZ10-siRNA silenced cell line was further incubated for 96 h with the corresponding extracted exosomes, containing a total protein concentration of 20 µg/µl. Cells treated only with siPOR-NeoFX transfection agent were used as negative controls.

Subsequently, the cells were washed twice with PBS, fixed with 96% cold ethanol for 30 minutes at 4°C and permeabilized for 15 minutes with 0.25% Triton X-100 in PBS when necessary. Then, the cells were treated with blocking solution for nonspecific sites with 5% normal serum in PBS for 1 h at room temperature and then incubated at 4°C with the primary anti-FZD10 (1:200; Abcam, Cambridge, UK), anti-ERK1/2 (1:200; Abcam, Cambridge, UK), anti-p-ERK1/2 (1:200; Abcam, Cambridge, UK), and anti-Ki-67 [Ki-67 Mib-1 Clone, mouse monoclonal antibody (DAKO)] overnight. The treated cells were then incubated with a specific green fluorescent-conjugated secondary IgG Alexa 488 (Invitrogen) (λ_abs,max_ 495 nm; λ_emi,max_ 517 nm) for 1 h and mounted using Prolong Gold Antifade reagent containing DAPI (λ_abs,max_ 345 nm; λ_emi,max_ 455 nm). Images were acquired with a Nikon Eclipse Ti2 fluorescence microscope and analyzed by using the interactive software installed on the machine. The images were acquired by exciting with Kr-Ar and Ar lasers fitted with 488 and 358 nm band-pass filters, respectively, for the FZD10, ERK, p-ERK, and Ki-67 green channel (488 nm) and for the DAPI blue channel (358 nm) at ×40 magnification. The fluorescence intensity was quantified by using an exposure time of 500 ms per acquisition for all the investigated samples.

### Bioinformatic and Statistical Analyses

Interactions among significant FZD10 and Ki-67 proteins were predicted with Search Tool for the Retrieval of Interacting Genes/Proteins (STRING) database version 11.0 (30, 31). Statistical analysis was performed with Sigma-Stat 3.1 software, applying one-way analysis of variance (ANOVA). Differences were regarded as statistically significant at p < 0.005 or p < 0.001. When mean equality among groups was rejected by the one-way ANOVA, the Holm–Sidak method was applied for the comparison. The Pearson correlation was applied to investigate correlations between the expression of Ki-67 in tissues and of FZD10 in exosomes.

## Results

### Increase of FZD10 Protein Level in Plasma-Derived Exosomes and Tissues

Exosomes were isolated from plasma of healthy donors and GC- or CRC-affected subjects by multiple centrifugation steps. After their physical characterization (**Figure S1, Supplementary Material**), the total protein content was extracted from exosomes, and the FZD10 expression level was studied by Western blotting in exosomes isolated from the plasma of 20 healthy donors, 26 CRC patients, and 20 GC patients (**Table 1**). ALIX and HSP-70 were also investigated as relevant marker proteins for exosomes. Semiquantitative evaluation of FZD10 expression level in the samples for each patient was carried out by means of densitometry analysis and by using the HSP-70 protein band for the normalization of the FZD-10 band.

**Figure 1:**
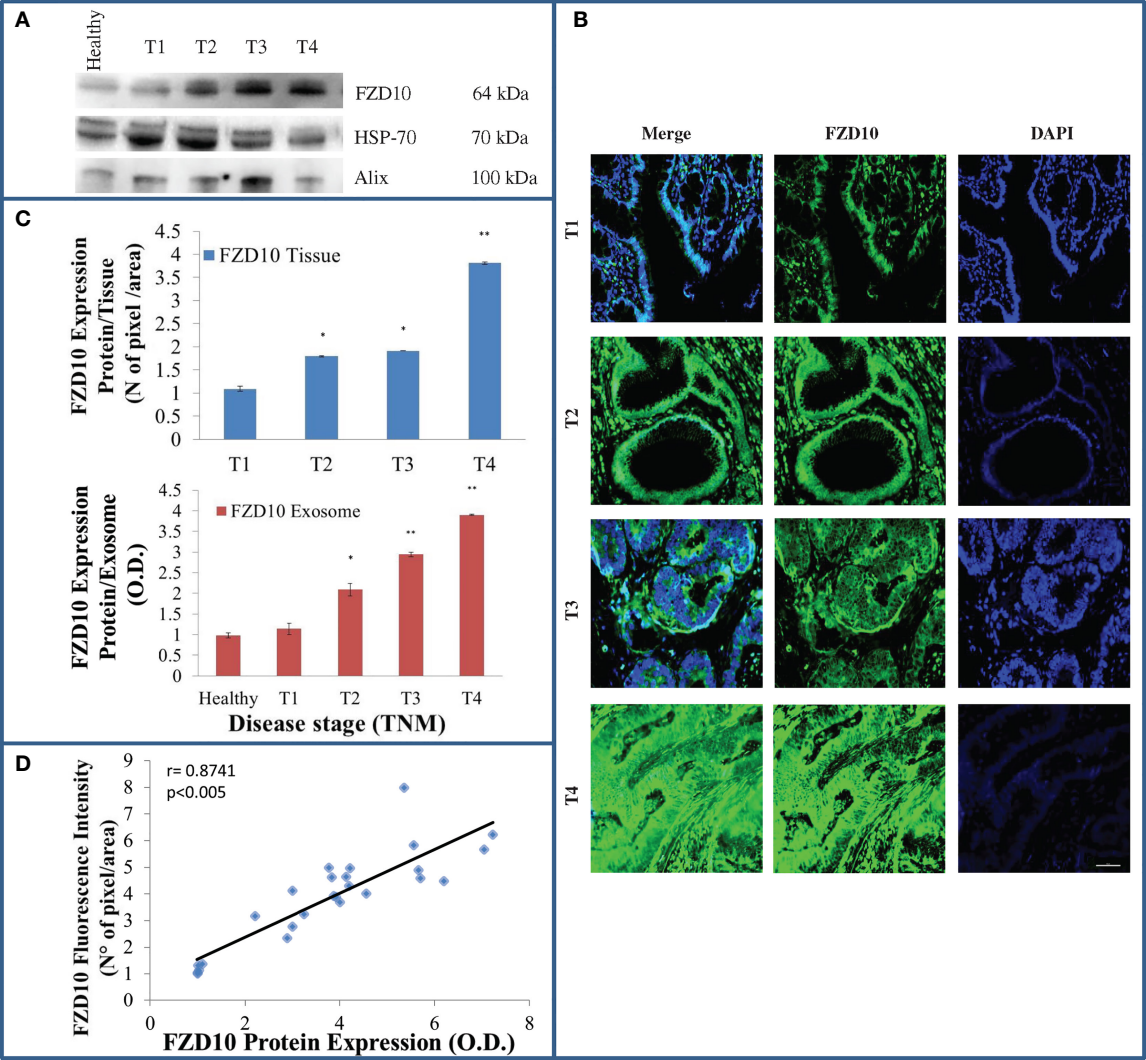
Evaluation of Frizzled (FZD)10 in exosomes isolated from plasma and corresponding tissue samples derived from (26) colorectal cancer (CRC) patients at different TNM stages. Representative Western blotting of FZD10 and the two marker proteins (HSP-70 and ALIX) in exosomes derived from (20) healthy donors and CRC patients **(A)**. Representative confocal microscopy images of CRC tissues for the detection of FZD10 by immunofluorescence. Blue channel: nuclei; green channel: labeled FZD10 and corresponding overlay. Scale bar, 50 µm. Magnification, ×20 **(B)**. Semiquantitative evaluation of FZD10 expression level in exosomes extracted from healthy donors and CRC patients by video-densitometry analysis of FZD10 bands on Western blotting. The HSP-70 protein band was used for the normalization of the FZD10 band for each subject. (*) p < 0.005 and (**) p < 0.001 *vs*. healthy donors (red bars). FZD10 expression levels, quantitatively evaluated as mean green fluorescence intensity index, in tissues by immunofluorescence (blue bars). (*) p < 0.005 and (**) p < 0.001 *vs*. CRC patients with stage T1 **(C)**. Pearson’s correlation between FZD10 expression levels in exosomes determined by Western blotting analysis and in the corresponding tissues determined by immunofluorescence analysis for each CRC-affected subject from stages T1 to T4. p < 0.005 **(D)**.

**Figure 1A** shows a representative Western blotting of FZD10 and two marker proteins in exosomes isolated from healthy subjects and CRC patients at different TNM stages. The semiquantitative analysis of the density of FZD10 bands on Western blotting indicated a significant increase (**Figure 1C**, red bars) in the FZD10 expression levels in CRC patients at different stages compared to healthy subjects, specifically, p < 0.005 for T2 and T3 *vs*. control and p < 0.001 for T4 *vs*. control.

FZD10 expression level was also investigated in the corresponding colon tissue samples by fluorescent immunohistochemical staining and imaging. Representative immunofluorescence images, recorded by confocal microscopy, of colon tissue samples derived from CRC patients at different TNM stages and the corresponding FZD10 expression levels, evaluated as mean green fluorescence intensity index, are reported in **Figures 1B, C** (blue bars), respectively. Imaging of the green fluorescent FZD10 in tissues revealed its presence in different cellular compartments of the tissue samples at the different TNM stages investigated. FZD10 expression was nuclear in CRC tissues at stage T1, while the green fluorescent signals of the overexpressed protein revealed its translocation into the cytoplasm and cell membranes in CRC tissues, indicating progressive evolution from stages T2 to T4 (**Figure 1B**).

The immunofluorescence analysis shows a statistically significant increase of the fluorescence intensity index (p < 0.005 *vs*. control cells) of FZD10 in CRC tissues at T2, T3 (p < 0.005 *vs*. healthy donors), and T4 (p < 0.001 vs. healthy donors) stages when compared to colon tissues of CRC patients at stage T1, in accordance with the results obtained by densitometry analysis performed on the total protein content extracted from exosomes. To investigate the occurrence of a relationship between the expression levels of FZD10 in exosomes isolated from plasma and in the corresponding tissues for each CRC patient, the Pearson correlation coefficient was calculated, as shown in **Figure 1D**. The value of the correlation coefficient was r = 0.8741, thus exceeding 0.7, indicating a strong positive correlation between the FDZ10 expression level in exosomes and in tissues.

**Figure 2:**
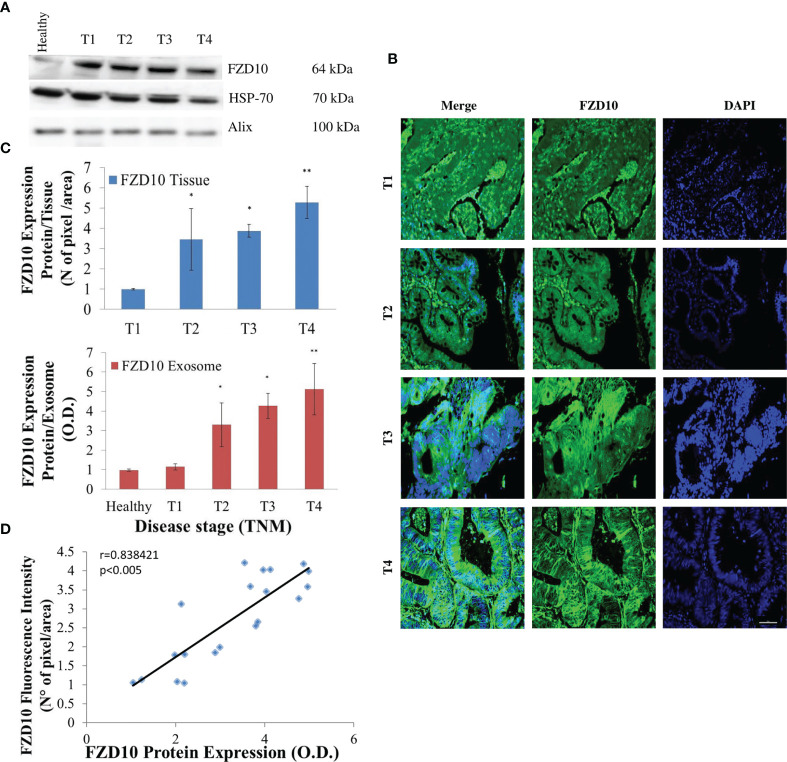
Evaluation of Frizzled (FZD)10 in exosomes isolated from plasma and corresponding tissue samples derived from (20) gastric cancer (GC) patients at different TNM stages. Representative Western blotting of FZD10 and the two marker proteins (HSP-70 and ALIX) in exosomes derived from (20) healthy donors and GC patients **(A)**. Representative confocal microscopy images of tissues for the immunofluorescence detection of FZD10. Blue channel: nuclei; green channel: labeled FZD10 and corresponding overlay. Scale bar, 50 µm. Magnification, ×20 **(B)**. Semiquantitative evaluation of FZD10 expression level in exosomes extracted from healthy donors and GC patients by video-densitometry analysis of FZD10 bands on Western blotting. The HSP-70 protein band was used for the normalization of the FZD10 band for each subject. (*) p < 0.005 and (**) p < 0.001 *vs*. healthy donors (red bars). FZD10 expression level, quantitatively evaluated as mean green fluorescence intensity index, in tissues by immunofluorescence (blue bars). (**) p < 0.001 *vs*. GC patients with stage T1 **(C)**. Pearson’s correlation between FZD10 expression levels in exosomes by Western blotting analysis and the corresponding tissues by immunofluorescence analysis for each subject. p < 0.005 **(D)**.

The same approach, including evaluation of FZD10 expression level in exosomes and in tissue for each patient, by Western blotting and immunofluorescence analysis, was applied in patients affected by GC at various stages of the disease. As in the study on CRC patients, both the Western blotting results and immunofluorescence data revealed a statistically significant (p < 0.001 *vs*. healthy donors) progressive increase of the FZD10 expression level in exosomes and tissues from patients at stage T1 to stages T2–T4, as compared to healthy subjects (**Figures 2A** **–C**). Also in this case, translocation of the FZD10 was observed, as it appeared localized in the nucleus in the tissues from T1 patients, while it persisted in the nucleus but spread to the cytoplasm in T2. As the severity of the disease increased, at stages T3 and T4, a complete translocation of the protein into the cytoplasm and membranes was observed (**Figure 2B**).

As in the case of CRC, also in GC, the expression levels of FZD10 both in exosomes and tissues from each affected subject were found to be strongly correlated (**Figure 2D**), the Pearson correlation value, r, being 0.8384.

### *In Silico* Prediction of a Link Between FZD10 and Ki-67 Interactions Mediated by MAPK3

**Figure 3:**
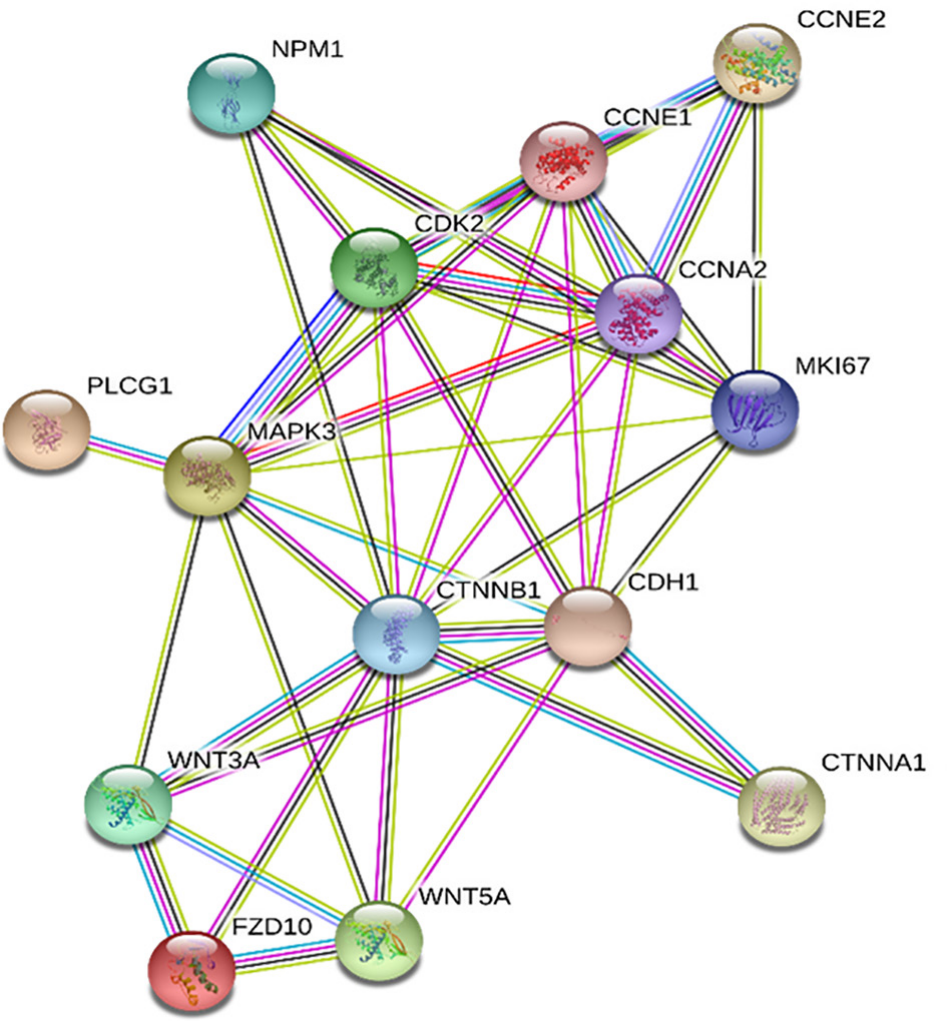
Frizzled (FZD)10 and Ki-67 protein interaction network by Search Tool for the Retrieval of Interacting Genes/Proteins (STRING). The network nodes are proteins; the edges represent the predicted functional associations based on seven different types of evidence including fusion evidence, neighborhood evidence, co-occurrence evidence, experimental evidence, text mining evidence, database evidence, and co-expression evidence. Color legend: □ curated databases; □ experimentally determined; □ co-expression; □ gene neighborhood; □ gene fusions; □ gene co-occurrence; □ text mining; □ protein homology

Prediction of the interacting proteins involved in the relationship between FZD10 and Ki-67, performed by using the STRING database, indicated that the activation of the canonical and noncanonical Wnt/calcium pathways was mediated by Wnt3A and Wnt5A. Moreover, MAPK3 (also known as ERK1/2) seemed to act as the core of the network, thus possibly representing the link between FZD10 and Ki-67 interaction (**Figure 3**).

### Significant Difference in the Expression Levels of ERK1/2 and Phospho-ERK1/2 in Tissues but Not in Exosomes

**Figure 4:**
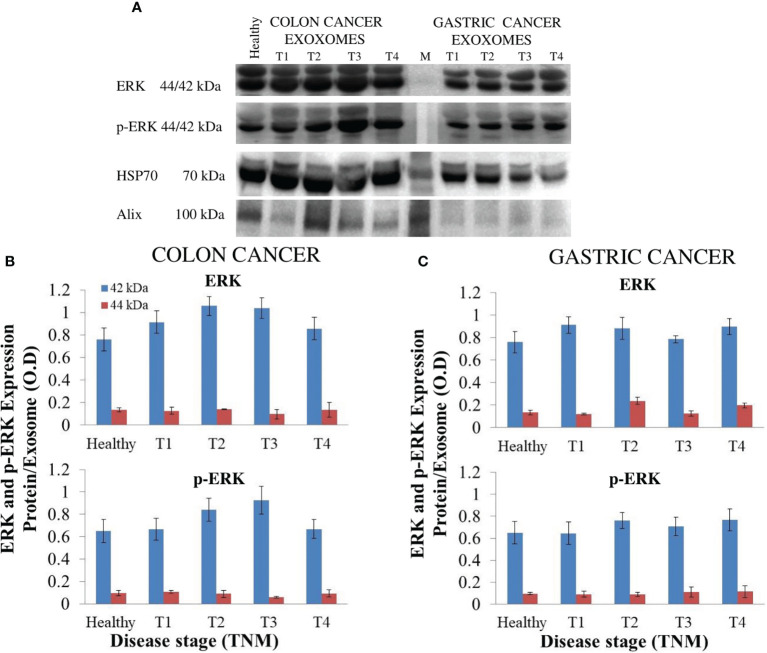
Evaluation of extracellular signal-regulated protein kinase (ERK)1/2 and p-ERK1/2 in exosomes derived from plasma of (20) healthy donors and (26) colorectal cancer (CRC) or (20) gastric cancer (GC) patients at different TNM stages. Representative Western blotting of ERK1/2, p-ERK1/2, and the two marker proteins (HSP-70 and ALIX) in exosomes **(A)**. Semiquantitative evaluation of ERK1/2 and p-ERK1/2 expression level in exosomes isolated by plasma of CRC **(B)** and GC **(C)** patients at different TNM stages by video-densitometry analysis of bands of ERK1/2 and p-ERK1/2 proteins on Western blotting performed by using distinct gels and membranes for the different proteins. Healthy donors were used as control. HSP-70 protein band was used for the normalization of the ERK1/2 and p-ERK1/2 bands for each subject **(B, C)**.

To achieve biological validation of the findings of the bioinformatic study, the expression level of ERK1/2 and phospho-ERK1/2 (p-ERK1/2) in the protein cargo extracted from exosomes derived from plasma of CRC and GC patients and of healthy donors was evaluated by Western blotting analysis (**Figure 4**). The semiquantitative analysis indicated no significant difference in the expression level of ERK1/2 and p-ERK1/2 in the exosomes derived from plasma of healthy volunteers and of the patients affected by CRC or GC at the different TNM stages (**Figure 4**).

**Figure 5:**
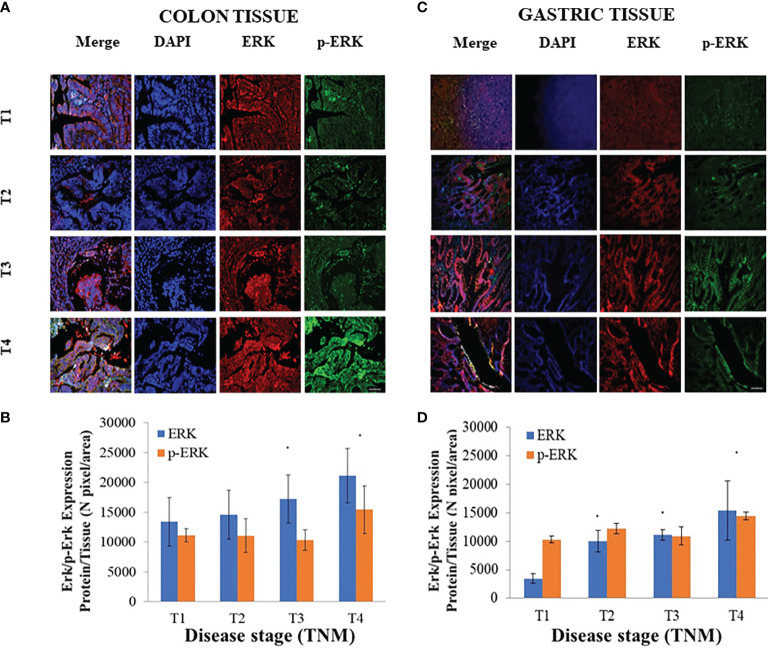
Evaluation of extracellular signal-regulated protein kinase (ERK)1/2 and p-ERK1/2 expression levels in tissues derived from (26) colorectal cancer (CRC) or (20) gastric cancer (GC) patients. Representative confocal microscopy images **(A, C)** for the detection of ERK1/2 and p-ERK1/2, along with ERK1/2 and p-ERK1/2 expression levels **(B, D)**, evaluated by fluorescence intensity index analysis, in CRC **(A, B)** and GC **(C, D)** tissues at different TNM stages. Blue channel: nuclei; green channel: labeled p-ERK1/2; red channel: labeled ERK1/2 and corresponding overlay. Scale bar, 50 µm; Magnification, ×20. (*) p < 0.005 *vs*. CRC or GC patients with stage T1 **(A–D)**.

Conversely, a significant increase (p < 0.005 *vs*. CRC or GC patients with T1 stage) in the expression levels of both the ERK1/2 and p-ERK1/2 proteins, evaluated by immunofluorescence analysis, was demonstrated in the CRC pathological tissues at the highest investigated TNM stage, namely, T4 (**Figures 5A, B**). For GC patients, while a significant increase of the expression tissue level of ERK1/2 was always observed in the tissues at T2, T3, and T4 stages, no modification of the p-ERK1/2 expression level was noted when progressing from cancer stages T1 to T3, thus this was significantly higher only at stage T4 as compared to stage T1 (**Figures 5C, D**).

### Strong Positive Correlation Between FZD10 in Exosomes and Ki-67 in Tissues

**Figure 6:**
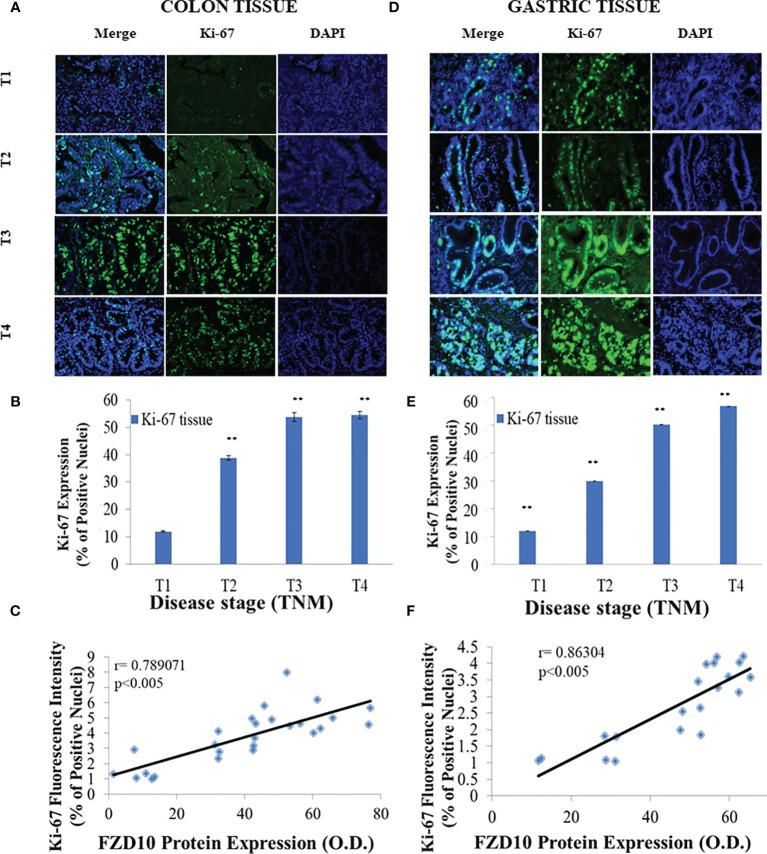
Evaluation of Ki-67 expression levels in tissues derived from (26) colorectal cancer (CRC) or (20) gastric cancer (GC) patients and correlation between Ki-67 in tissues and Frizzled (FZD)10 expression in exosomes. Representative confocal microscopy images for the detection of Ki-67+ nuclei (green) in tissues from CRC **(A)** or GC **(D)** patients at different TNM stages. Blue channel: nuclei; green channel: labeled Ki-67 and corresponding overlay. Scale bar, 50 µm; Magnification, ×20 **(A, D)**. Ki-67 expression levels, quantitatively evaluated by immunofluorescence intensity index, in CRC **(B)** and GC **(E)** tissues at different TNM stages. (**) p < 0.001 *vs*. T1 subject **(B, E)**. Pearson’s correlations between expression levels of FZD10 in exosomes evaluated by Western blotting analysis and Ki-67 in CRC **(C)** or GC **(F)** tissues evaluated by immunofluorescence analysis p < 0.005 *vs*. stage T1 **(C, F)**.

Expression levels of Ki-67 were evaluated by immunofluorescence investigation (**Figures 6A** **–E**). Results indicated a statistically significant increase (p < 0.001 *vs*. T1 patient donors) in Ki-67 expression in pathological tissues, starting from stage T2 in CRC patients (**Figures 6A, B**) and from stage T1 in GC patients (**Figures 6D, E**).

The possible relationship existing between the expression levels of FZD10 in exosomes, evaluated by Western blotting analysis (**Figures 1C** and **2C**; red bars), and of Ki-67 in the corresponding tissues, estimated by immunofluorescence analysis (**Figures 6B, E**), was calculated for CRC and GC patients by applying the Pearson statistics. Correlation coefficient (r) values of 0.789 and 0.863 were obtained for CRC and GC patients, respectively, thus demonstrating a strong positive correlation between the expression levels of the FZD10 in exosomes and of Ki-67 stained in the tissues (**Figures 6C, F**).

**Figure 7:**
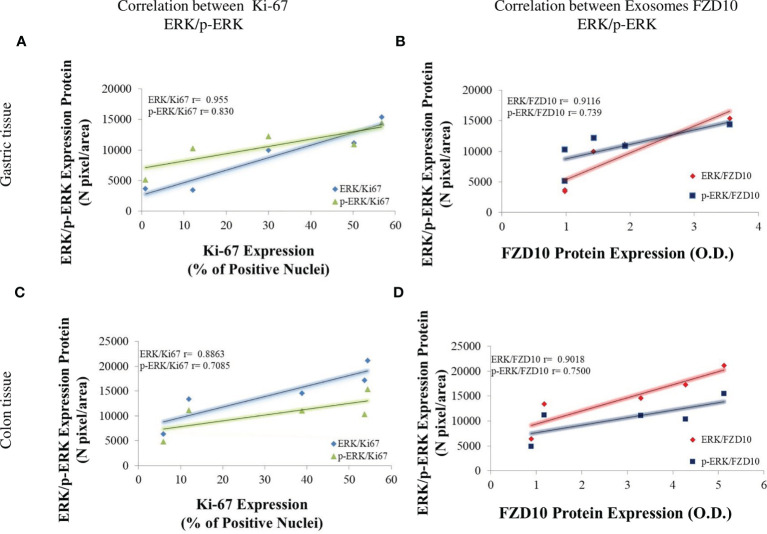
Pearson’s correlation of all evaluated markers. Pearson’s correlations between expression levels of Ki-67 *vs*. extracellular signal-regulated protein kinase **(**ERK)1/2 (blue line) and Ki-67 *vs*. p-ERK (green line) by immunofluorescence analysis in gastric cancer (GC) **(A)** or colorectal cancer (CRC) **(C)** tissues at different TNM stages **(A, C)**. Pearson’s correlations between expression levels of ERK1/2 *vs*. Frizzled (FZD)10 (red line) and p-ERK *vs*. FZD10 (dark blue line). Expression levels of FZD10 were evaluated in exosomes by Western blotting analysis, while expression levels of ERK and p-ERK were evaluated in tissues by immunofluorescence analysis of GC **(B)** or CRC **(D)** patients at different TNM stages **(B, D)**. In all the plots, each point was obtained as average value of all enrolled CRC or GC patients enrolled at the same TNM stage (from T1 to T4). p < 0.005.

Finally, there was a strong correlation between the expression levels of ERK/p-ERK and Ki-67 in tissues and between the expression levels of FZD10 in exosomes and of ERK/p-ERK in tissues in CRC and GC patients at the different TNM stages (**Figure 7**).

### FZD10-mRNA Silencing in Cell Culture

**Figure 8:**
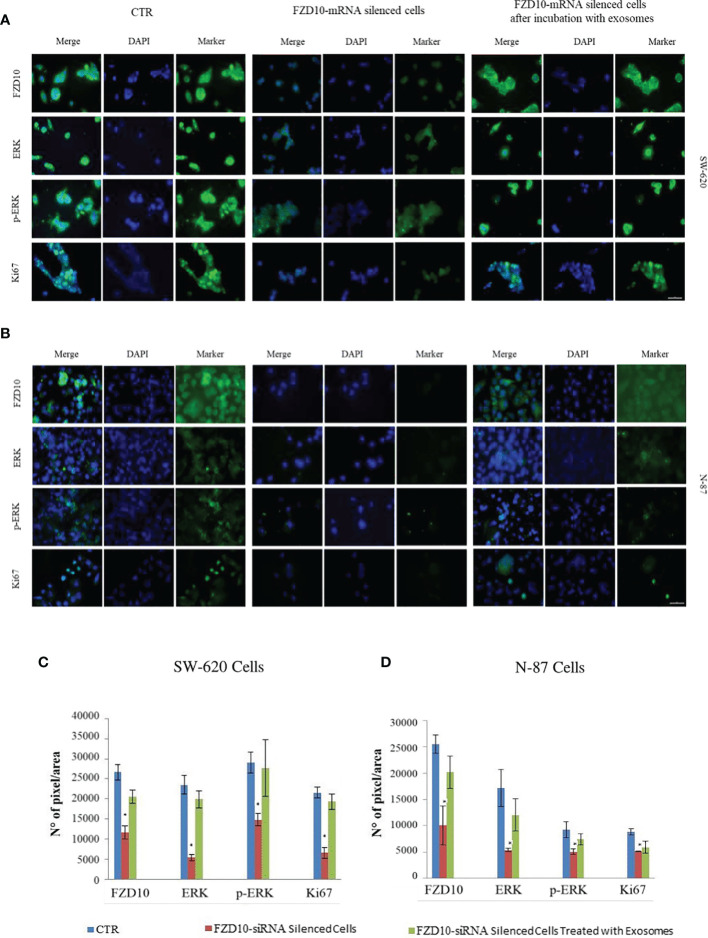
Detection of Frizzled (FZD)10, extracellular signal-regulated protein kinase (ERK), p-ERK, and Ki-67 by immunofluorescence confocal microscopy in fixed SW620 **(A)** and N87 **(B)** cells. Confocal microscopy images and immunofluorescence by mean intensity index of FZD10-mRNA silenced SW620 **(A, C)** and FZD10-mRNA silenced N87 **(B, D)** cells before and after incubation with corresponding exosomes. For each cell line, cells treated only with the si-PORT-NeoFX transfection agent were used as negative control (CTR). Blue channel: nuclei; green channel: labeled respectively FZD10, ERK, p-ERK, and Ki-67; merge: overlay of blue and green channels. Scale bar, 20 µm. (*) p < 0.001.

To further investigate the role of FZD10 on tumor proliferation, we silenced FZD10 in SW620 and N87, CRC and GC cells, respectively, and analyzed pERK1/2 and Ki-67 expression by confocal microscopy investigation before and after cell treatment with exosomes. The immunofluorescence intensity index shows a statistically significant reduction (p < 0.001) of pERK1/2 and Ki-67 in the silenced cells compared to the corresponding controls for both cell lines (**Figure 8**). Incubation with exogenous exosomes for 96 h restores the expression level of both pERK1/2 and Ki-67, suggesting a role of FZD10 in cancer cell proliferation *via* pERK1/2 (**Figure 8**).

## Discussion

FZD10, one of the 10 proteins belonging to the G protein receptor family, is known to be involved in the Wnt signaling cascade and upregulated in several malignancies including CRC and GC (1, 11, 21, 22, 32). For example, Nagayama et al. (18) highlighted the effective role of FZD10 in the progression of CRCs by analyzing the expression patterns of FZD10 in colonic polyps, primary CRCs, and metastatic liver lesions. More recently, we described the expression and localization of FZD10 protein in tissues of patients with CRC, melanoma, and GC at different disease stages (20). The involvement of the FZD10 in CRC is also suggested by The Cancer Genome Atlas (TGCA) that pointed out the occurrence of mutation or overexpression of the gene encoding FZD10 in CRC (33). Consistently, Terasaki et al. (13) revealed the upregulation of FZD10-mRNA in primary CRC and suggested its possible involvement in carcinogenesis through activation of the Wnt–β-catenin–TCF signaling pathway. The presence of both the FZD10-protein and FZD10-mRNA was also detected in exosomes extracted from culture medium of different gastroenteric tract cancer cell lines. Restoration of their expression levels as well as the cell viability was observed after incubation of each silenced cell line with the exosomes extracted from culture medium of the same untreated cell line (26).

Recently, investigations into tumor development have taken into account stimulation not only by autocrine signaling but also by paracrine actions stimulating carcinogenesis exerted by the extracellular vesicles (EVs), such as exosomes. Several studies indicated that cancer-derived exosomes can play a relevant role in the progression of CRC and GC (34–37). Evidence has been reported on a role of exosomes in the promotion of the EMT (38–41). In particular, FZD10-delivering exosomes were also indicated to trigger the EMT in a normal epithelial colon cell line, together with an increase of proteins, such as vimentin and c-Myc, shown to be overexpressed and involved in the mechanisms of cell migration, cell adhesion, and proliferation (27).

On the basis of all these above-described experimental evidence that highlighted an active involvement of FZD10-delivering exosomes in carcinogenesis and evolution of CRC and other cancers of the gastroenteric tract, the present clinical study reports follow-up on our previous studies concerning the FZD10 upregulation in the exosomes isolated from the plasma of CRC and GC patients (21). The aim is to further validate FZD10 as a novel plasma biomarker candidate for early diagnosis and prognosis of CRC and GC. Therefore, to probe the possible clinical and predictive significance of FZD10 delivered by plasma exosomes in the diagnosis and prognosis of CRC and GC, the present study investigated, for the first time, the relationship between the expression levels of the FDZ10 in plasma-derived exosomes and of Ki-67 in tissues from the colon or stomach of CRC and GC patients (at different TNM stages). Very few studies on the correlation between expression of specific markers, delivered by exosomes or EVs, and the expression of Ki-67, in tumor tissues, have been reported. For example, Wang et al. (42) showed that epidermal growth factor receptor (EGFR)-positive EVs are effective diagnostic and prognostic markers of glioma. They demonstrated that expression of the EGFR in serum EVs can accurately differentiate high-grade and low-grade glioma patients, and EGFR in EVs were positively correlated with Ki-67 in the tumor tissue (42). Very recently, Zadkam et al. (43) found a strong positive correlation between the cell proliferation antigen, Ki-67, and specific exosome markers (CD9 and CD63) in CRC patients by isolating exosomes from solid tissues.

As a first step in the study, exosomes were isolated in plasma from healthy subjects and patients affected by CRC and GC at different TNM stages and characterized by DLS and TEM analysis and ζ-potential measurements. All the isolated exosome samples exhibited a spherical shape, negative surface charge, and average hydrodynamic diameters lower than 150 nm in accordance with our previously reported procedures and already published data (**Figure S1, Supplementary Material**) (21). A strong correlation between the expression levels of FZD10 in plasma-derived exosomes from patients affected by CRC and GC and the expression levels of the same protein in the tissues from the same subjects was firstly obtained (**Figures 1** and **2**). This strong correlation can be explained by taking into account that exosomes are vesicles generated from parts of cytoplasmic membranes, according to their biogenesis mechanism (44). If membranes and cytoplasm of cells in the tissues of oncological patients, at TNM stages higher than T1, are FZD10 enriched (**Figures 1B** and **2B**), consequently, the corresponding secreted exosomes will be characterized by higher expression levels of FZD10 than that in exosomes from healthy subjects and CRC and GC patients at stage T1 (20).

A preliminary bioinformatic prediction of proteins that are activated by FZD10 prior to Ki-67 and that, therefore, act as signaling intermediates in the crosstalk between FZD10 and Ki-67, performed by using the STRING database (30, 31), indicated that ERK1/2 was at the core of the signaling network (**Figure 3**). The MAPK/ERK pathway and ERK1/2, known to be involved in the development of human cancers, were found to contribute to the proliferation of cancer cells. High expression levels of p-ERK1/2 were observed in several human cancers (10, 36). Therefore, the present study focused on the evaluation of expression levels of ERK-1/2 and p-ERK-1/2 in exosomes and tissues from CRC and GC patients. No significant differences in ERK-1/2 and p-ERK-1/2 levels between exosomes derived from healthy subjects and those affected by CRC or GC were observed (**Figure 4**). Conversely, significant differences in the expression of these proteins in the tissues of oncological patients at the highest investigated TNM stages were found when compared with tissues derived from CRC or GC patients with stage T1 (**Figure 5**). A strong positive correlation was found between FDZ10 in exosomes and Ki-67 in tissues, FZD10 in exosomes and ERK/p-ERK in tissues, and Ki-67 and ERK/p-ERK both in tissues (**Figures 6** and **7**). The possible occurrence of an actual relationship in tissues between FZD10 or ERK/p-ERK1/2 and the clinicopathological factor Ki-67 is a key issue that needs to be closely assessed to understand the role of these tumor-activated proteins in CRC and GC patients. For example, Vicent et al. (45) analyzed, by immunohistochemistry, the expression of p-ERK1/2 that was found to be activated in the tumoral conditions. They investigated its relationship with several clinicopathological factors, including Ki-67, in patients with advanced non-small-cell lung cancer. No Ki-67 expression was found to correlate, in the tissue specimens analyzed, with activated ERK1/2 (cytoplasmic or nuclear) as detected by immunohistochemistry (45).

Conversely, in oral squamous cell carcinoma, Mishima et al. (46) demonstrated a statistical correlation between ERK and Ki-67, during the histologic grade study. In Hodgkin lymphomas and related lymphomas, Tian et al. (47) observed that EZH2 expression correlated with the proliferation rate, as assessed by Ki-67 staining, and they suggested its oncogenic role in these cancers. In human salivary gland mucoepidermoid carcinoma, Handra-Luca et al. (10) proved that ERK-1/ERK-2 phosphorylation was associated with increased Ki-67, thus suggesting that the ERK-1/ERK-2 pathway is active in salivary gland mucoepidermoid carcinoma, and this activation is associated with a more aggressive tumor behavior and a higher proliferative activity.

The above-described results suggest that the correlation between Ki-67 and FZD10 may be due to the Ki-67 activation, mediated by MAPK3 and induced by exogenous FZD10, then delivered to the recipient cells by exosomes. One of the proteins activated by the Wnt pathway is phospholipase C (PLC) that induces the activation of MAPK3 (ERK1/2 and p-ERK1/2), as well as the consequent downstream activation of CDK2 (**Figure 4**), and, finally, the activation of Ki-67 through phosphorylation and an increased cell proliferation (48). This hypothesis is further corroborated by the outcome of an additional silencing *in vitro* study on metastatic colon cancer cell line SW620 and a metastatic gastric cancer cell line N-87 (**Figure 8**). Furthermore, the *in vitro* experiments confirmed the effective role of FZD10 delivered by exosomes in activating cell proliferation and restoring expression levels of ERK, p-ERK. and Ki-67. A significant reduction of cell viability was observed in FZD10-siRNA silenced SW620 and N87 cells (26), and a complete recovery of the viability and expression of ERK, p-ERK, and Ki-67 was obtained after the administration of exosomes derived from the same untreated cell lines.

Therefore, the data obtained by bioinformatic and experimental studies suggested the occurrence of a possible interplay between the canonical and noncanonical Wnt pathways and MAPK signaling induced by FZD10-delivering exosomes in CRC and GC. On the other hand, several studies demonstrated that the activation of both canonical and noncanonical Wnt signaling pathways, depending on the alterations of the Wnt pathway components and their functions, is indispensable for the CRC onset, progression, and metastasis (6, 8–10). Furthermore, several researchers recently identified the crosstalk between the Wnt and ERK pathways (19–23, 49). In the context of intestinal tumorigenesis, the relationship between the Wnt/β-catenin and MAPK signaling pathways was investigated by Jeong et al. (5), who reported that the stimulation of the Wnt/β-catenin pathway leads to the activation of the MAPK pathway through the RAS stabilization. Therefore, they suggested that Wnt/β-catenin and MAPK signaling is synergized in a combined effort to promote transformation in CRC (5). Furthermore, Kim et al. (50) developed a plausible model based on the integration of experimental reports and established basic mathematical models of each pathway (the Wnt/β-catenin and MAPK signaling pathways) to demonstrate the occurrence of a positive feedback loop in the Wnt/ERK crosstalk pathway in CRC. This positive feedback loop induces maximal and sustained ERK activity and thereby upregulates β-catenin/TCF (50).

Our findings demonstrated that the FZD10, located in the metastatic cells and delivered by the secreted exosomes, plays a relevant role during the metastatic evolution, survival, and proliferation of cancer cells. In this study, the active involvement of FZD10 in the development of CRC and GC was assessed and further confirmed in cell cultures and in the patients’ tissues. The FZD10 expression level in plasma exosomes of healthy donors and CRC and GC patients at different TNM stages was demonstrated to be strongly correlated to the Ki-67 expression level in the corresponding tissues from the colon or stomach. The results obtained from plasma-derived exosomes and the corresponding tissues from oncological subjects confirmed the link between FZD10 and Ki-67 mediated by MAPK3, namely, ERK1/2 and p-ERK1/2, in agreement with the bioinformatic prediction.

The absence of significant changes in the expression levels of ERK-1/2 and p-ERK-1/2 in exosomes, as compared to their high expression levels in tissues, allowed us to reasonably infer that the observed increase of ERK and p-ERK expression levels in the pathological tissues depends on the activation of the pathway involved in their overexpression. This influences the mechanisms of Ki-67 phosphorylation and hence activation. Therefore, the present experimental findings, taken together, including the outcome of the *in vitro* study on SW620 and N87 cells, suggest that exogenous FZD10, delivered by exosomes, is able to activate the canonical and noncanonical Wnt pathways, inducing the activation of Ki-67 proliferation mediated by MAPK3. Further investigations are needed to gain a deeper understanding of the complex network of molecular mechanisms involved. In short, the correlations obtained between Ki-67 expression in tissues and FZD10 in exosomes, from the same patient, as well as FZD10 expression in both tissues and exosomes, hold great promise for the application of FZD10 as a novel prognostic and diagnostic biomarker for CRC and GC. It may serve to promote the development of plasma-based assays (liquid biopsy) for noninvasive diagnosis and staging of CRC and GC, supporting a more timely management of CRC and GC.

## Data Availability Statement

The raw data supporting the conclusions of this article will be made available by the authors, without undue reservation.

## Ethics Statement

The studies involving human participants were reviewed and approved by The study was conducted according to the guidelines of the Declaration of Helsinki, and approved by the Ethics Committee of Istituto Tumori “Giovanni Paolo II” (protocol code 329 and date of approval 29/07/2019). The patients/participants provided their written informed consent to participate in this study.

## Author Contributions

MS and ND contributed to the conceptualization. MS, FR, ND, SC, GS, EF, and PP contributed to the methodology. EF and NC contributed the software. GS, MC, and GG contributed to the validation. MS, ND, FR, and RA contributed to the formal analysis. MS, FR, SC, PP, and AC contributed to the investigation. GG contributed to the resources. GS, RA, MS, ND, and MC contributed to data curation. MS and ND contributed to writing–original draft preparation. MS, ND, MC, and GG contributed to writing–review and editing. MS, GG, and RA contributed to the supervision. GG contributed to project administration and funding acquisition. All authors contributed to the article and approved the submitted version.

## Funding

This article was funded by Ministero della Salute.

## Conflict of Interest

The authors declare that the research was conducted in the absence of any commercial or financial relationships that could be construed as a potential conflict of interest.

## Publisher’s Note

All claims expressed in this article are solely those of the authors and do not necessarily represent those of their affiliated organizations, or those of the publisher, the editors and the reviewers. Any product that may be evaluated in this article, or claim that may be made by its manufacturer, is not guaranteed or endorsed by the publisher.
