# Supplementary figures and images for: Exosome Released FZD10 Increases Ki-67 Expression via Phospho-ERK1/2 in Colorectal and Gastric Cancer
Source: Front Oncol. 2021 Sep 23;11:730093. doi: 10.3389/fonc.2021.730093 (PMC8522497; doi:10.3389/fonc.2021.730093)

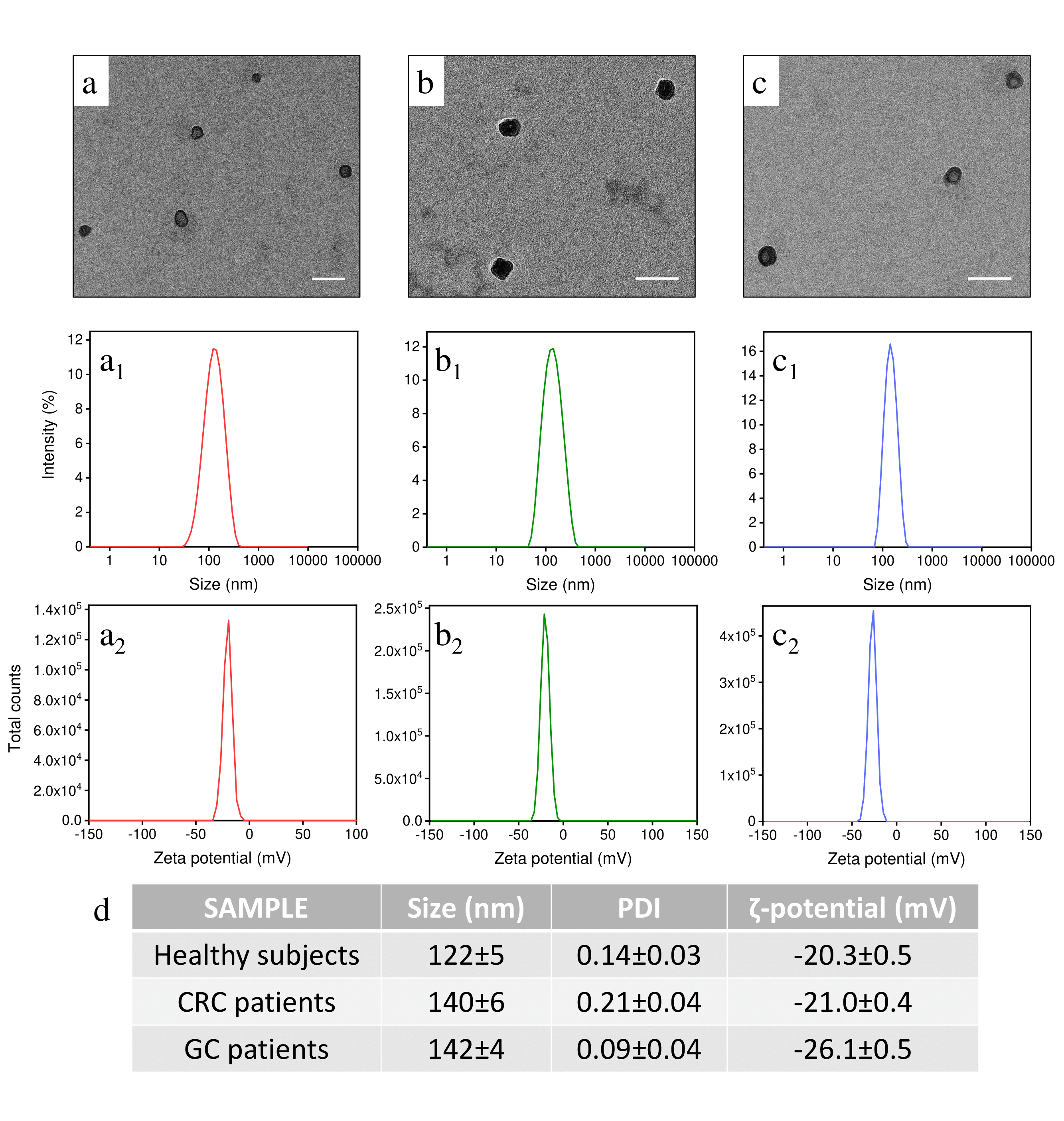

Supplement: Supplementary file 1 [file Image_1.tiff]

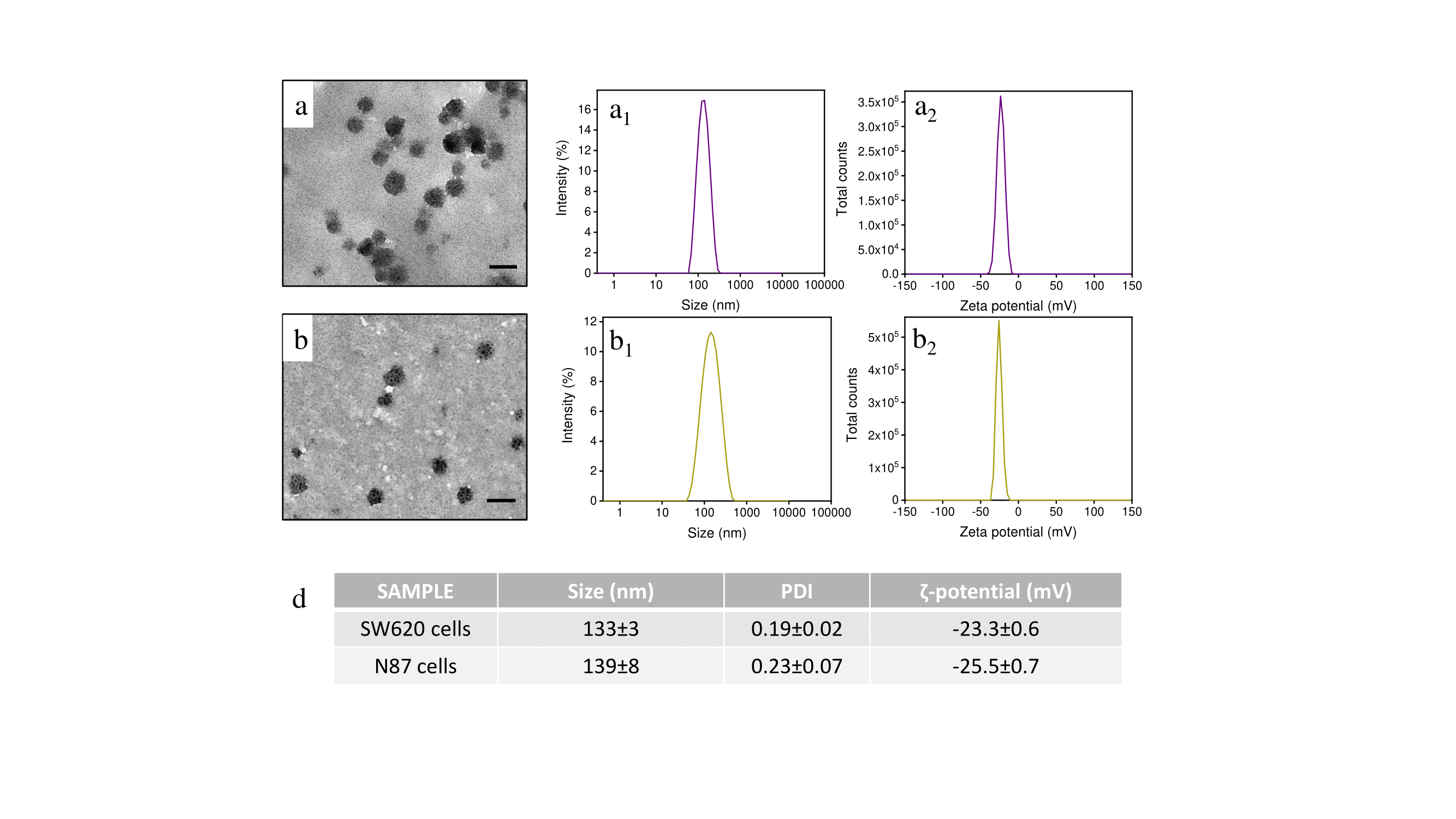

Supplement: Supplementary file 2 [file Image_2.tiff]
